# Epidemiology of Maxillofacial Injuries in the Swords of Iron War: Insights From a National Registry

**DOI:** 10.1111/edt.13081

**Published:** 2025-06-13

**Authors:** Nir Tsur, Dmitry Kotovich, Dean Dudkiewicz, Tomer Talmy, Irina Radomislensky, Adi Givon, Israel Trauma Group, H. Bahouth, M. Bala, A. Bar, A. Braslavsky, D. Czeiger, D. Fadeev, A. L. Goldstein, I. Grevtsev, G. Hirschhorn, I. Jeroukhimov, A. Kedar, Y. Klein, A. Korin, B. Levit, U. Neeman, I. Schrier, A. D. Schwarz, W. Shomar, D. Soffer, O. Yaslowitz, I. Zoarets, Avi Benov, Yael Arbel, Mor Rittblat, Shachar Shapira

**Affiliations:** ^1^ Israeli Defense Forces Medical Corps Tel Hashomer, Ramat Gan Israel; ^2^ Department of Otolaryngology‐Head and Neck Surgery Rabin Medical Center Petah Tikva Israel; ^3^ Department of Plastic and Reconstructive Surgery Hadassah Hebrew University Medical Centre Jerusalem Israel; ^4^ Department of Military Medicine and “Tzameret,” Faculty of Medicine The Hebrew University of Jerusalem Jerusalem Israel; ^5^ Division of Anesthesia, Intensive Care & Pain Management Tel‐Aviv Sourasky Medical Center Tel‐Aviv Israel; ^6^ Gertner Institute of Epidemiology and Health Policy Research Sheba Medical Center Ramat Gan Israel; ^7^ Department of Periodontology Sheba Medical Center Tel Hashomer Israel; ^8^ Department of Emergency and Disaster Management, School of Public Health Faculty of Medical and Health Sciences, Tel Aviv University Israel; ^9^ Management Wing Sheba Medical Center Ramat Gan Israel

**Keywords:** epidemiology, maxillofacial injuries, prehospital, trauma, war

## Abstract

**Background:**

Maxillofacial injuries (MFI) in warfare present significant challenges due to the concentration of vital structures in the facial region and the high‐energy mechanisms of injury. This study analyzes the epidemiology, severity, and outcomes of MFI during the Swords of Iron conflict using data from the Israeli National Trauma Registry.

**Methods:**

A retrospective analysis was conducted on casualties from October 7, 2023 to August 1, 2024. Injury characteristics, surgical interventions, intensive care unit (ICU) admissions, and mortality rates were assessed. Logistic regression identified predictors for severe head injury and surgical intervention.

**Results:**

Of 1654 casualties, 324 (19.6%) sustained MFI, predominantly from explosions (85.2%). Fractures were associated with higher ICU admission rates (60.6% vs. 20.0%, *p* < 0.0001) and greater surgical intervention requirements (65.4% vs. 36.4%, *p* = 0.0001) than soft tissue injuries. Orbital fractures (59.6%) were the most common and strongly correlated with severe head injury (OR 9.33, CI 4.29–21.54, *p* < 0.001). Zygomatic (OR 8.47), maxillary (OR 5.81), and mandibular fractures (OR 12.61) significantly predicted the need for surgery, whereas orbital fractures often did not. Airway management was required in 26.5% of MFI cases, significantly higher than in other injuries (12.6%, *p* < 0.001). The overall mortality rate was low, with 7.7% in the fracture group and 0.9% in the soft tissue injury group.

**Conclusion:**

MFI in combat settings demands specialized trauma care, particularly for fractures requiring surgical intervention and intensive care. The high prevalence of orbital fractures and their association with severe head injuries highlight the need for early recognition and intervention despite their being less prone to surgical correction. These findings can inform trauma care protocols to optimize management and outcomes in future conflicts.

## 1 Introduction

Maxillofacial injuries (MFI) present a significant challenge in both civilian and military settings due to the complex anatomy and vital structures within the facial region [1, 2]. In modern conflicts, the incidence of MFI has increased due to advancements in high‐energy munitions and the protective effects of body armor and helmets, which reduce injuries to the trunk and head while leaving the face more vulnerable [3, 4].

Combat‐related MFI is often associated with high‐energy trauma mechanisms, including explosions, gunshot wounds, and shrapnel impacts, which lead to intricate fractures and significant soft tissue damage. Historically, military management of MFIs has prioritized rapid stabilization and surgical intervention, particularly for injuries compromising airway patency or associated with other severe injuries, such as traumatic brain injury (TBI) [5, 6]. These injuries not only present immediate life‐threatening implications but also lead to significant long‐term morbidity, including functional, psychological, and cosmetic impairments [5, 7].

The Swords of Iron War (SOI) is an armed conflict between Israel and Hamas, which began in October 2023. This study aims to evaluate the incidence, patterns, and outcomes of MFIs among military personnel during this war. By analyzing injury characteristics, mechanisms, and the need for surgical intervention, this study seeks to provide valuable insights into managing MFIs in combat settings.

## 2 Materials and Methods

The study was approved by an Institutional Review Board (IRB) as required for such studies [8]. The manuscript was written and edited according to the STROBE statement guidelines [9].

This study extracted data from hospitalized Israeli trauma casualties recorded in the Israeli National Trauma Registry (INTR) from the SOI between October 9, 2023, and May 31, 2024. Hence, it does not encompass all casualties from this ongoing conflict. The INTR records [10] are detailed in the supplement. In brief, the extracted data from the INTR comprised the date of injury, demographic, vital signs, and clinical information, including etiology, severity index, intensive care unit (ICU) admission, length of stay (LOS) in the hospital, and discharge destination. Alveolar bone fractures were considered dental trauma, as this condition alone does not typically require hospitalization or surgical intervention [11]. The diagnoses were classified using the Abbreviated Injury Scale (AIS), which assigns a severity score to each injury on a scale from 1 to 6: 1—Minor (superficial injuries such as small lacerations or contusions), 2—Moderate (more significant but not life‐threatening injuries, such as simple fractures), 3—Serious (injuries requiring immediate medical intervention, such as multiple rib fractures), 4—Severe (life‐threatening injuries with a high probability of survival, such as complex fractures with significant blood loss), 5—Critical (life‐threatening injuries with uncertain survival, such as severe brain trauma), and 6—Fatal (injuries incompatible with life).

Statistical analysis included Chi‐square tests and Fisher's exact tests for group comparisons as appropriate. Logistic regression analyses were employed to assess binary outcomes. Odds ratios (OR) were computed to estimate the likelihood of a specific outcome associated with each predictor, with 95% confidence intervals (CI) and *p*‐values. The concordance index measured the model's predictive performance. Two univariate analyses were conducted for a preliminary identification of significant predictors. The first was employed to identify predictors for surgical intervention among casualties with maxillofacial injuries, and the second to analyze potential variables associated with AIS + 3 head injury. Multivariable logistic regression was then utilized to adjust for confounding factors and ascertain independent predictors. Variables for the analyses included those with clinical relevance and statistical significance in univariate analysis or those identified in previous literature as potential predictors. All statistical analyses were performed using the SAS Software version 9.4 (SAS, Cary, NC). A *p*‐value less than 0.05 was considered statistically significant.

## 3 Results

### 3.1 Baseline Characteristics of MFI Compared to Other Sites of Injury

Table 1 summarizes the characteristics of subjects suffering from MFI compared to subjects with other sites of injury. A total of 1654 injured soldiers, comprising 324 (19.6%) with MFIs and 1330 (80.4%) with injuries to other sites, were included in the study. Groups were predominantly male (99.1% vs. 98.7%, respectively, *p* = 0.78), with a median age of 23 in both groups. The primary mechanism of injury (MOI) was explosions (85.2% vs. 65.1%) followed by gunshot wounds (8.6% vs. 19.2%), both significantly different between the groups (*p* < 0.001). Additionally, the heart rate (over 130BPM) and the Glasgow Coma Scale (GCS) were significantly different between the groups (*p* < 0.001) at Emergency Room (ER)‐admission. Preliminary data on dental injuries were diagnosed in 25.9% of head and neck injured soldiers; these results will be further described in a different work by this group.

**TABLE 1:** Comparative analysis of MFI versus non‐MFI from Swords of Iron War.

Variable	All maxillofacial injuries (*n* = 324)	Other injuries (*n* = 1330)	*p*
Sex: Male	321 (99.1%)	1312 (98.7%)	0.8
Age—Median (IQR)	23 (19–55)	23 (18–59)	
MOI			
Explosion	276 (85.19%)	866 (65.11%)	**< 0.001**
GSW	28 (8.64%)	265 (19.92%)	
Heart rate (+130 BPM)	26 (8.3%)	39 (3.04%)	**< 0.001**
GCS			
3–8	30 (9.6%)	51 (3.9%)	**< 0.001**
9–14	17 (5.5%)	30 (2.3%)	
15	265 (84.9%)	1226 (93.8%)	
Disposition from ER			
Surgery	93 (28.70%)	344 (25.86%)	**< 0.001**
ICU	40 (12.35%)	58 (4.36%)	
Ward	189 (58.33%)	921 (69.25%)	
Death	2 (0.62%)	6 (0.45%)	
Total ICU Admission	107 (33.02%)	211 (15.86%)	**< 0.001**
LOS			
0–1	81 (25.31%)	397 (30.10%)	**0.003**
2–6	115 (35.94%)	548 (41.55%)	
7–13	50 (15.63%)	162 (12.28%)	
14+	74 (23.13%)	212 (16.07%)	
ISS			
Mild 1–8	145 (44.75%)	816 (62.87%)	**< 0.0001**
Moderate 9–14	59 (18.21%)	267 (20.57%)	
Severe 16–24	55 (16.98%)	110 (8.47%)	
Critical 25+	65 (20.06%)	105 (8.09%)	
Hospital discharge			
Death	10 (3.09%)	23 (1.73%)	0.1
Home	191 (58.95%)	877 (65.94%)	
Rehab	120 (37.04%)	423 (31.80%)	

*Note:* Bold values are significant values and the corresponding *p*‐values are already provided in the tables.

Abbreviations: BPM, Beats per minutes; ER, Emergency Department; GCS, Glasgow Coma Scale; ICU, Intensive Care Unit; IQR, Interquartile range; ISS, Injury Severity Score; LOS, Length of Stay; MOI, Mechanism of Injury.

Significant differences were noted in patient disposition from the ER, with subjects in the MFI group admitted directly to the ICU more than the other injuries group (12.4% vs. 4.4%, *p* < 0.001); and had a significantly higher prevalence of ICU admission during hospitalization (33.02% vs. 15.86%, *p* < 0.001). Length of stay (LOS) and Injury Severity Score (ISS) were significantly higher for the MFIs group (*p* < 0.01 for both). The MFIs group showed a trend (*p* = 0.06) toward higher discharge to rehab (37.0% vs. 31.8%) and a higher death rate (3.1% vs. 1.7%) compared to the other injury group.

### 3.2 Maxillofacial Fractures Compared to Soft Tissue Injuries

Out of the 324 subjects with MFI, only 104 (32.1%, all male) had maxillofacial fractures, with the rest (220, 98.6% male) having only soft tissue injuries. Table 2 summarizes the characteristics of both groups. The main MOI was explosions (81.7% vs. 86.8%) followed by gunshot wounds (10.6% vs. 7.7%), both significantly not different between the groups (*p* = 0.36). Penetrating injuries presented in both groups (77.9% vs. 80.9%, *p* = 0.53). The GCS significantly differed (*p* < 0.001) at ER admission.

**TABLE 2:** Characteristics of MFI of fracture‐related versus soft tissue injuries.

Variable	Maxillofacial fractures (*n* = 104)	Soft tissue injuries (*n* = 220)	*p*
Sex: Male	104 (100.00%)	217 (98.64%)	0.6
Age‐Median (IQR)	22 (19–46)	23 (19–55)	0.9
MOI			
Explosion	85 (81.73%)	191 (86.82%)	0.4
GSW	11 (10.58%)	17 (7.73%)	
Penetrating Injury	81 (77.88%)	178 (80.91%)	0.5
GCS			
3–8	22 (22.9%)	8 (3.7%)	**< 0.001**
9–14	7 (7.3%)	10 (4.6%)	
15	67 (69.8%)	198 (91.67%)	
ER discharge destination			
Surgery	52 (50.00%)	41 (18.64%)	**< 0.001**
ICU	20 (19.23%)	20 (9.09%)	
Ward	32 (30.77%)	157 (71.36%)	
Death	0 (0.00%)	2 (0.91%)	
ICU Admission	63 (60.58%)	44 (20.00%)	**< 0.001**
LOS			
0–1	8 (7.92%)	73 (33.33%)	**0.001**
2–6	26 (25.74%)	89 (40.64%)	
7–13	25 (24.75%)	25 (11.42%)	
14+	42 (41.58%)	32 (14.61%)	
ISS			
Mild 1–8	18 (17.31%)	127 (57.73%)	**< 0.001**
Moderate 9–14	16 (15.38%)	43 (19.55%)	
Severe 16–24	24 (23.08%)	31 (14.09%)	
Critical 25+	46 (44.23%)	19 (8.64%)	
Surgery (any site)	68 (65.38%)	80 (36.36%)	**< 0.001**
System with AIS + 3 injury			
Head	53 (50.96%)	31 (14.09%)	**< 0.001**
Face	51 (49.04%)	4 (1.82%)	**< 0.001**
Chest	28 (26.92%)	27 (12.27%)	**0.001**
Abdomen	7 (6.73%)	9 (4.09%)	0.3
Spine	3 (2.88%)	5 (2.27%)	0.7
Upper Extremity	12 (11.54%)	19 (8.64%)	0.4
Hospital Discharge			
Rehab	54 (51.92%)	66 (30.00%)	**< 0.001**
Home	40 (38.46%)	151 (68.64%)	
Death	8 (7.69%)	2 (0.91%)	

*Note:* Bold values are significant values and the corresponding *p*‐values are already provided in the tables.

Abbreviations: AIS, abbreviated injury scale; ER, Emergency Department; GCS, Glasgow Coma Scale; ICU, Intensive Care Unit; IQR, Interquartile range; ISS, Injury Severity Score; LOS, Length of Stay; MOI, Mechanism of Injury.

Significant differences were noted in patient disposition from ER, with subjects with fractures admitted directly to the OR or ICU more than the soft tissue‐only group (50.0% vs. 18.6%; 19.2% vs. 9.1%, respectively *p* < 0.001 for both). The fractures group had a higher ICU admission during hospitalization (60.6% vs. 20.0%, *p* < 0.001) and surgery at any point during hospitalization (65.4% vs. 36.4%, *p* < 0.001). LOS, ISS, and AIS + 3 were significantly higher for the fractures group (*p* < 0.01 for all). The fracture group had significantly higher discharge to rehab (51.9% vs. 30.0% *p* < 0.01) and a higher death rate (7.7% vs. 0.9%, *p* < 0.01) compared to the soft tissue‐only group.

### 3.3 Type of Fractures

**FIGURE 1:**
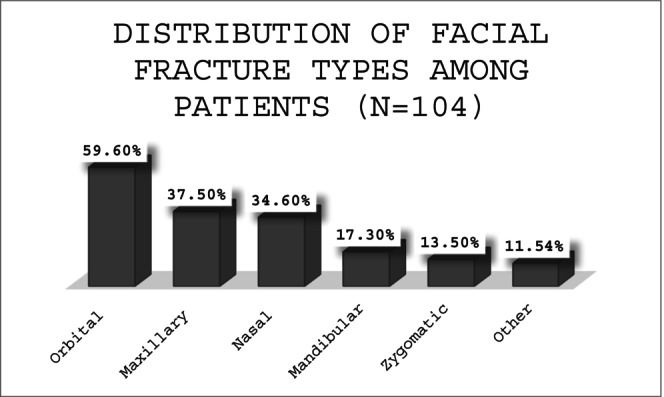
The bar chart represents the distribution of different types of facial fractures among 104 subjects with maxillofacial injuries. Orbital fractures were the most common (59.62%), followed by maxillary (37.50%) and nasal fractures (34.62%). Other types of fractures were less frequent, such as mandibular (17.31%), zygomatic (13.46%), and other unspecified fractures (11.54%).

Among the 104 subjects analyzed, various types of facial fractures were identified and subsequently treated. Orbital fractures were the most common, with 62 subjects (59.62%) presenting with this type of injury. Maxillary fractures were observed in 39 subjects (37.50%), followed by nasal fractures in 36 (34.62%). Mandibular fractures were present in 18 subjects (17.31%), while zygomatic fractures were less frequent, occurring in 14 subjects (13.46%). A smaller group, consisting of 12 subjects (11.54%), sustained other types of facial fractures. Out of the total cohort, 19 subjects (18.27%) required surgical intervention to address their facial bone fractures (Figure 1).

### 3.4 Airway Intervention

**FIGURE 2:**
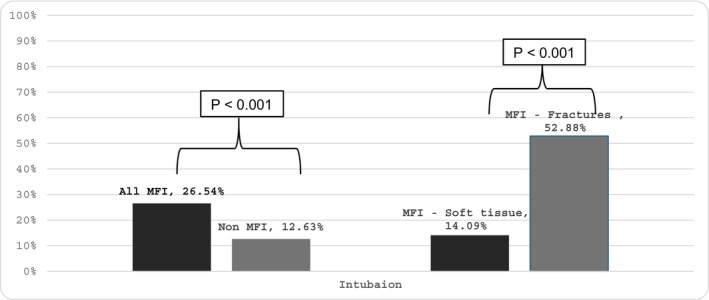
The bar chart illustrates the comparative distribution of airway intervention performed across various patient groups: MFIs versus Non‐MFI, and within the MFIs subgroup, soft tissue injuries versus fractures. Each bar represents the percentage of subjects in each category.

Figure 2 summarizes the distribution of the airway intervention procedures (i.e., intubation or tracheostomy) across four groups: MFIs, other injuries, MFIs—soft tissue only, and MFIs—fractures only. Significant differences were noted between the MFIs versus other injuries group and between the MFIs—soft tissue only versus MFIs—with fractures groups. The MFIs group had a significantly higher distribution of airway intervention at the prehospital or ER admission (26.54% vs. 12.63%, *p* < 0.001) compared to other injuries. Among the MFIs subgroups, the fractures‐only group had a significantly higher distribution of airway intervention versus the soft tissue‐only group (52.88% vs. 14.09%, *p* < 0.001).

### 3.5 Logistic Regressions Assessing the Association of Severe Head Injury and Surgical Intervention

**FIGURE 3:**
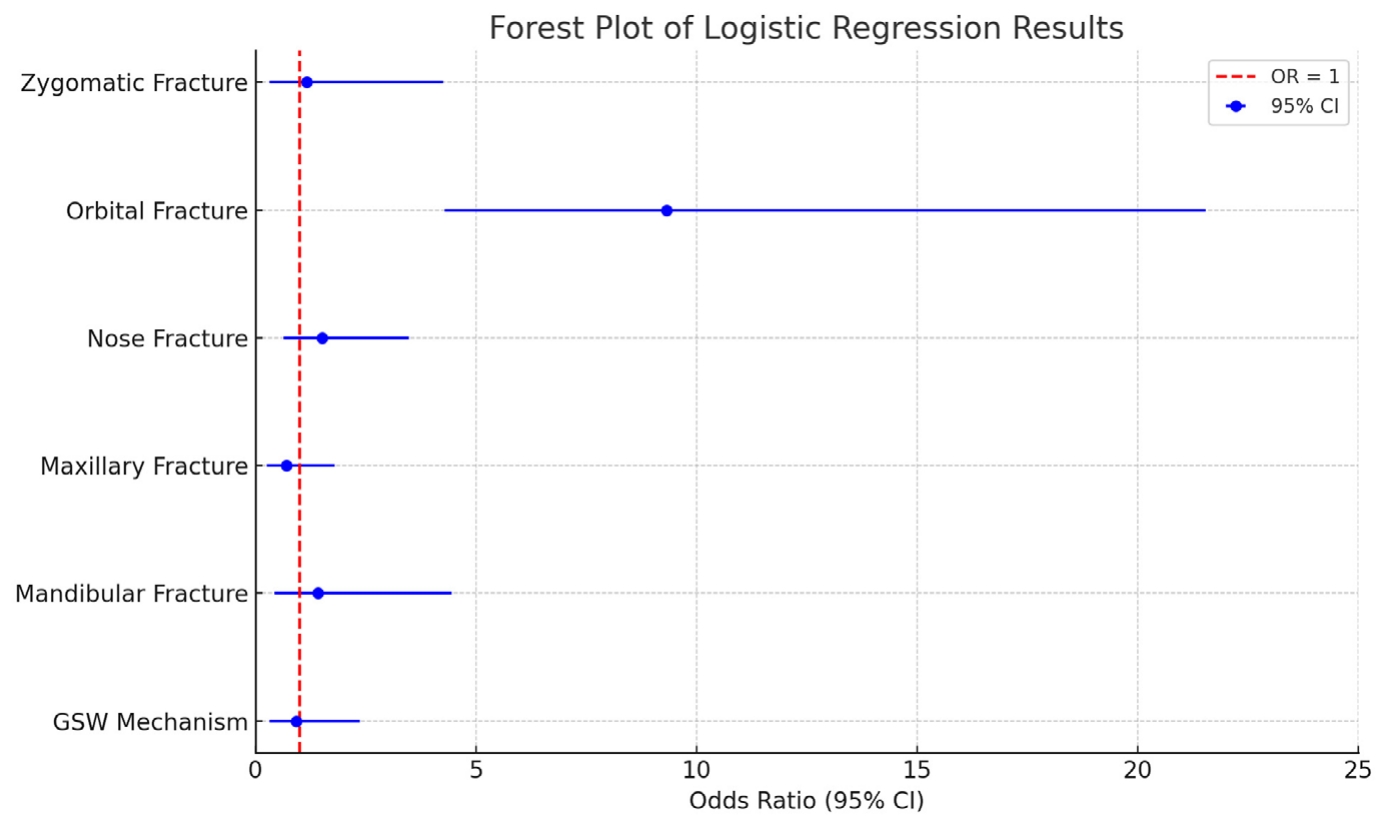
The plot visualizes the odds ratios (OR) with 95% confidence intervals (CI) for variables predicting AIS + 3 head injury. The footnote explains the significance of the OR values and the reference line at OR = 1, which indicates no effect. This format is designed to present.

**FIGURE 4:**
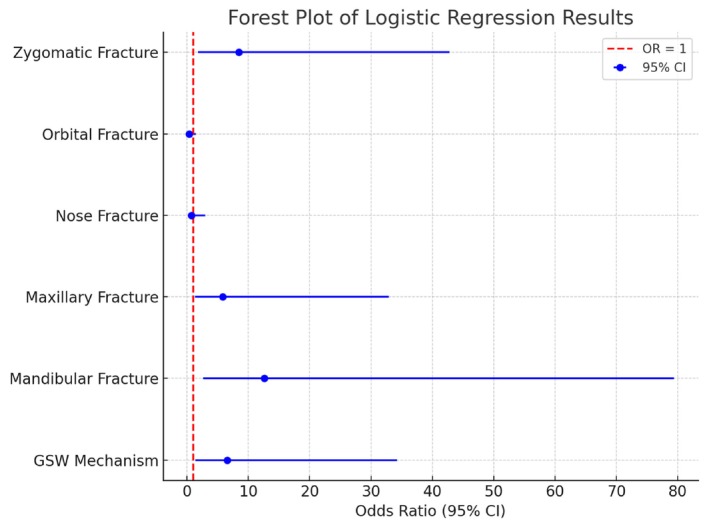
The plot visualizes the odds ratios (OR) with 95% confidence intervals (CI) for variables predicting Surgical Intervention. The footnote explains the significance of the OR values and the reference line at OR = 1, which indicates no effect. This format is designed to present the results of the logistic regression analysis.

Multivariate logistic regression analysis assessing the risk for severe head injury revealed that only orbital fractures (OR 9.33 [CI 4.29–21.54], *p* < 0.001) were associated with a significantly higher likelihood of severe head injury. In contrast, all other facial bone fractures showed no significant impact (Figure 3). The severity of injuries in other body regions (AIS ≥ 3) and the MOI, including penetrating trauma, were not significantly associated with either surgical intervention or severe head injury. As for surgical intervention, the regression analysis showed zygomatic fractures (OR 8.47 [CI 1.79–42.81], *p* < 0.01), maxillary fractures (OR 5.81 [CI 1.34–32.86], *p* = 0.026), and mandibular fractures (OR 12.61 [CI 2.64–79.36], *p* < 0.001) were significantly associated with an increased likelihood of requiring surgical intervention, while orbital (OR 0.37 [CI 0.08–1.47], *p* = 0.16) and nasal fractures (OR 0.74 [CI 0.15–3.0], *p* = 0.68) did not (Figure 4).

## 4 Discussion

Penetrating MFI, especially in military settings, poses unique challenges, including increased risk of infection, extensive tissue damage, and the need for complex surgical reconstruction [12]. This study analyzes MFIs from the first year of the SOI war, highlighting epidemiological patterns, injury severity, and outcomes. Among 1654 casualties, 19.6% sustained MFIs, mostly involving soft tissue damage. Maxillofacial fractures were observed in 32.1% of MFI cases, with 7.9% caused by penetrating trauma—higher than in civilian settings where blunt trauma predominates [13]. This pattern is consistent with prior military conflicts, reflecting a rise in injury rates due to increased use of high‐energy explosives [3].

Consistent with prior military conflicts, including those involving the IDF and U.S. forces, MFIs are increasing, likely due to the widespread use of high‐energy explosives [14]. Similarly, past analyses of the Israel Defense Forces during prior conflicts show an increasing trend in MFI, with rates of 6.4% in the Second Lebanon War (2006) [15] and 17.9% from 1997 to 2020 [5], likely attributable to the increase and widespread use of high‐energy munitions and explosive devices [16].

Airway interventions were significantly more common in MFI cases (26.5% vs. 12.6%; *p* < 0.001), especially among those with fractures (52.9%). This highlights the need for improved training and protocols for managing complex facial trauma in prehospital settings. These findings align with previous studies highlighting the high incidence of airway interventions in MFI subjects [17, 18]. The decision to establish a definitive airway in the prehospital environment presents unique challenges due to several factors. First, while injuries to the limbs and torso are more common and familiar to prehospital providers, MFI is comparatively rare, potentially limiting experience and confidence in managing these complex cases. Second, using sedatives to facilitate definitive airway placement poses significant risks. Sedatives can exacerbate airway compromise by relaxing supportive structures and worsening obstruction, while inadequate sedation can hinder the completion of a definitive airway [19]. Third, despite their often‐devastating appearance, some MFI can be managed conservatively until the patient reaches a hospital, where a controlled environment with specialized staff and advanced equipment can allow for a safer and more definitive airway intervention. These findings highlight the critical need for enhanced training and the development of evidence‐based prehospital protocols tailored to the management of MFI.

Additionally, there was a notably high prevalence of orbital fractures, affecting 59.62% of MFI subjects (Figure 1). This rate is significantly higher than those typically reported in the literature, where orbital fractures comprise approximately 18%–25% of facial fractures [20, 21, 22]. Moreover, it aligns with a long‐standing trend of increasing combat‐related ocular injuries since 1967. During the Six‐Day War (1967) [23, 24], the ocular injury rate was 5.6%, rising to 6.7% in the Yom Kippur War (1973) [25], and 6.8% in the First Lebanon War. The rate further increased to 8% in the Second Lebanon War (2006) and peaked at 11% during Operation “Cast Lead” (2008, unpublished data) [26, 27, 28, 29]. The elevated rate of these injuries likely results from the specific injury mechanisms in this conflict compared to past conflicts, particularly the extensive use of explosive devices [30]. Orbital fractures are complex injuries that often necessitate intervention to prevent long‐term complications such as vision impairment and facial deformity [17]. The high prevalence observed here highlights the critical need for protective strategies to safeguard the orbital region in combat environments [18].

Interestingly, orbital fractures were not significant predictors of the need for surgical intervention, while zygomatic, maxillary, and mandibular fractures were found to be associated with the need for surgical intervention with odds ratios (OR) for surgical intervention of 8.47 (CI 1.79–42.81, *p* < 0.01), 5.81 (CI 1.34–32.86, *p* = 0.0267), and 12.61 (CI 2.64–79.36, *p* < 0.001), respectively. As with other facial fractures, the complexity of these injuries, combined with associated risks such as compromised airway patency or severe cosmetic deformities, often necessitates surgical intervention [19, 31, 32]. In contrast, orbital fractures do not always require immediate surgery, as many can be managed non surgically, particularly when there are no acute changes in orbital volume [33]. Mandibular fractures can be treated by closed fixation (intermaxillary fixation), open fixation, or an external fixator according to fracture characteristics and blood supply. The high correlation found between mandibular fractures and surgical intervention can be explained by the mechanism of injury, which results in favorable architecture for surgery [34].

Orbital fractures were the only injury significantly associated with an increased risk for severe head injury, with an OR of 9.33 [CI 4.29–21.54], *p* < 0.001. This link between orbital fractures and severe head injury was shown before, with subjects suffering from orbital roof and lateral wall fractures found to have an increased risk for severe head injury, with up to 65% of them suffering severe head injury [35]. It highlights the interrelated nature of orbital fractures and severe head trauma in high‐energy injury scenarios [36]. In combat environments, where the likelihood of multiple injuries is elevated, clinicians must maintain a high index of suspicion for head trauma in subjects presenting with specific types of facial fractures [37]. Early identification and intervention for severe head injuries are critical in improving patient outcomes and preventing long‐term neurological complications [38].

The findings from this study have important implications for the clinical management of MFI in combat settings. Advancements in body armor have shifted injury patterns toward exposed areas like the face. The high MFI burden, especially orbital and mandibular fractures, highlights the urgent need for better facial protection and improved triage protocols. The high prevalence of orbital fractures and the significant role of penetrating trauma suggest that current protective measures may be inadequate, necessitating the development of more effective maxillofacial protective equipment [39]. Additionally, identifying specific fractures that predict the need for surgical intervention can improve the triage process, helping prioritize subjects requiring immediate surgical care [40]. Furthermore, our findings demonstrate a significantly longer length of stay (LOS) for MFIs compared to other injuries (23.1% vs. 16.1% for hospitalizations exceeding 14 days; *p* < 0.01) and for MFIs with fractures compared to soft‐tissue injuries (41.6% vs. 14.6%; *p* < 0.01). These results highlight the increased demand on hospital resources, particularly in managing complex fractures, and emphasize the critical need for improved protective measures to prevent such injuries in combat settings. Future research should focus on refining surgical techniques, improving rehabilitation strategies for subjects with complex MFI, and exploring the long‐term outcomes of these injuries to understand better their impact on quality of life [41]. Such advancements could significantly reduce morbidity, mortality, and injury severity.

This study has several limitations. First, it lacks data on prehospital treatment (other than airway intervention) and the use of protective gear (e.g., body armor or helmets), particularly in mortality cases involving MFI. Second, the absence of imaging data on the specific location or pattern of MFIs limits the ability to classify injuries more precisely and assess the effectiveness of protective equipment, such as ballistic goggles. Third, the data collection period may not fully capture all casualties from the conflict. Fourth, the study's retrospective design and reliance on registry data may introduce selection bias and limit the generalizability of the findings [42]. Finally, the lack of long‐term follow‐up data is a notable limitation, as it prevents an assessment of long‐term functional outcomes and complications. Future research should incorporate long‐term follow‐up to improve management strategies and better understand the lasting impact of MFIs.

This study highlights the unique and severe nature of MFIs sustained during the SOI and the increasing prevalence of such injuries in modern warfare. The findings underscore the need for targeted interventions, comprehensive management approaches, and advancements in protective strategies. Specifically, improving military protective gear—such as the development of lightweight facial protection—could help reduce the incidence and severity of head, face, and neck injuries. Additionally, enhancements in emergency trauma care, including optimized triage systems and rapid evacuation protocols, are crucial for improving survival and functional outcomes. Future research should focus on refining these strategies to better prevent and manage MFIs in combat environments.

## Author Contributions

N.T.: study concept and design, data acquisition, data analysis and interpretation, drafting of the manuscript, critical revision for important intellectual content. D.K.: data acquisition, drafting of the manuscript, critical revision of the manuscript for important intellectual content. D.D.: drafting of the manuscript, critical revision of the manuscript for important intellectual content. T.T.: drafting of the manuscript, critical revision of the manuscript for important intellectual content. I.R.: statistical analysis, data analysis and interpretation. A.G.: statistical analysis. I.T.G.: data acquisition and critical support. A.B.: study supervision, drafting and critical revision of the manuscript. Y.A.: drafting of the manuscript, critical revision for important intellectual content. M.R.: study concept and design, data analysis and interpretation, drafting of the manuscript, critical revision of the manuscript for important intellectual content. S.S.: study supervision, critical revision of the manuscript for important intellectual content.

## Ethics Statement

The study was approved by the Sheba Medical Center Institutional Review Board (IRB) (SMC 5138–18).

## Conflicts of Interest

The authors declare no conflicts of interest.

## Data Availability

The authors confirm that the data supporting the findings of this study are available within the article and its supplementary materials.

## Appendix

Israel Trauma Group: H. Bahouth, M. Bala, A. Bar, A. Braslavsky, D. Czeiger, D. Fadeev, A. L. Goldstein, I. Grevtsev, G. Hirschhorn, I. Jeroukhimov, A. Kedar, Y. Klein, A. Korin, B. Levit, U. Neeman, I. Schrier, A. D. Schwarz, W. Shomar, D. Soffer, O. Yaslowitz, I. Zoarets.
